# Three Novel Downstream Promoter Elements Regulate MHC Class I Promoter Activity in Mammalian Cells

**DOI:** 10.1371/journal.pone.0015278

**Published:** 2010-12-13

**Authors:** Namhoon Lee, Shankar S. Iyer, Jie Mu, Jocelyn D. Weissman, Anat Ohali, T. Kevin Howcroft, Brian A. Lewis, Dinah S. Singer

**Affiliations:** 1 Experimental Immunology Branch, National Cancer Institute, National Institutes of Health, Bethesda, Maryland, United States of America; 2 Cellular, Molecular, Developmental Biology and Biophysics, NIH-Johns Hopkins University, Bethesda, Maryland, United States of America; 3 Molecular Biology Institute, University of California Los Angeles, Los Angeles, California, United States of America; 4 Division of Cancer Biology, National Cancer Institute, Bethesda, Maryland, United States of America; 5 Metabolism Branch, National Cancer Institute, Bethesda, Maryland, United States of America; George Mason University, United States of America

## Abstract

**Background:**

MHC class I transcription is regulated by two distinct types of regulatory pathways: 1) tissue-specific pathways that establish constitutive levels of expression within a given tissue and 2) dynamically modulated pathways that increase or decrease expression within that tissue in response to hormonal or cytokine mediated stimuli. These sets of pathways target distinct upstream regulatory elements, have distinct basal transcription factor requirements, and utilize discrete sets of transcription start sites within an extended core promoter.

**Methodology/Principal Findings:**

We studied regulatory elements within the MHC class I promoter by cellular transfection and *in vitro* transcription assays in HeLa, HeLa/CIITA, and tsBN462 of various promoter constructs. We have identified three novel MHC class I regulatory elements (GLE, DPE-L1 and DPE-L2), located downstream of the major transcription start sites, that contribute to the regulation of both constitutive and activated MHC class I expression. These elements located at the 3′ end of the core promoter preferentially regulate the multiple transcription start sites clustered at the 5′ end of the core promoter.

**Conclusions/Significance:**

Three novel downstream elements (GLE, DPE-L1, DPE-L2), located between +1 and +32 bp, regulate both constitutive and activated MHC class I gene expression by selectively increasing usage of transcription start sites clustered at the 5′ end of the core promoter upstream of +1 bp. Results indicate that the downstream elements preferentially regulate TAF1-dependent, relative to TAF1-independent, transcription.

## Introduction

Transcription of genes by RNA polymerase II is a highly regulated process that requires the integration of multiple signaling pathways in order to generate a level of expression appropriate for a given set of environmental and cellular conditions. An important component of this regulation is the specific interactions between transcription factors and promoter DNA sequences that result in the assembly of the transcription initiation machinery [1]–[6] A diverse array of transcription factor binding sites located upstream of the major transcription start sites (TSS) reflect the abundance and complexity of regulatory interactions [7]. A similar complexity exists in the structures of core promoters – defined as the minimal length of DNA necessary to direct accurate transcription by RNA polymerase II (Pol II) [8]–[14].

The structures of core promoters vary but some features have contributed to our understanding of their function. In many promoters, TATA boxes and Inr elements function to establish a transcriptional start site [15]. A recently described class of elements, located downstream of the classical core promoter elements, has added another level of complexity to the core promoter architecture. These include the downstream promoter element (DPE) [8], [16], the downstream core element (DCE) [17], XCPE1 [18], XCPE2 [19] and the motif ten element (MTE) [20]. Like the TATA box, downstream elements, are constrained spatially within the core promoter architecture. For example, the DPE is centered at approximately +30 bp relative to the transcriptional start site. Disruption of spacing between the DPE and DCE classes of downstream elements and the transcriptional start site abrogates transcription [8], [11], [17]. These data imply that the trans-acting factors that interact with the downstream elements are equally constrained. Furthermore, based on their differing sequences, one would expect to find different factors interacting with them. Indeed, this is the case: the TFIID components, TAF6/TAF9, make direct contact with the DPE [21], [22]. In contrast, the TFIID component, TAF1, makes direct contact with the DCE in a sequence-specific manner [8], [15], [21], [23]. The sequence-specificity of the DCE and DPE extends beyond the DNA-binding components of TFIID, where DPE-specific transcription requires additional factors [23].

As the site of assembly of the transcription initiation machinery, the core promoter serves as a molecular platform to integrate regulatory signals delivered by upstream silencer and enhancer elements to appropriately adjust the level of promoter activity [2], [4], [6], [24]–[26]. The core promoter of major histocompatibility complex (MHC) class I genes provides an excellent model for genes subject to complex regulatory signals: MHC class I genes are constitutively expressed, but the relative levels of expression vary dramatically among different tissues, from very high in lymphoid tissues to exceedingly low in the nervous system and germ line [27]–[29]. Superimposed on its tissue-specific regulation, MHC class I expression is dynamically modulated by hormones, cytokines, and other inflammatory agents. For example, γ-interferon (IFN) increases class I transcription, whereas thyroid-stimulating hormone (TSH) represses it [30]–[32]. Thus, class I gene expression is subject to two distinct regulatory pathways: constitutive tissue-specific levels of transcription are established by a set of tissue specific factors that maintain homeostatic activity; cytokine- and hormone-specific factors superimpose a dynamic regulation of transcription.

The level of transcription for any given gene depends on the integration of its different regulatory pathways at the core promoter. Two related mechanisms exist that allow the core promoter to dictate these levels. First, some core promoters recruit distinct transcriptional machinery under different conditions. For example, IFN induced expression of MHC class I genes is mediated by CIITA, a non-DNA-binding co-activator that interacts with constitutively expressed RFX and ATF trans-acting factors already bound at the RFX/CRE site [33]–[38]. CIITA-mediated transcription bypasses the requirement for TAF1, a component of the TFIID general transcription factor that is necessary for class I transcription under constitutive conditions, suggesting that distinct transcriptional machineries are recruited to the class I promoter under different conditions [36].

Second, in some cases, core promoter regions differ in their pattern of transcription start sites (TSS). In yeast, it has been reported that the his3 promoter initiates transcription at two distinct TATA elements that are differentially utilized under constitutive or activated conditions [39]. Recent genome-wide analyses have reported that the majority of genes initiate transcription at multiple sites distributed over the core promoter region [40]. Many of these promoters reside in ATG deserts that constitute a novel sub-class of promoters [41]. Among these is the promoter of the MHC class I gene, PD1, which initiates transcription at multiple sites across an extended region of over 100 bp. Similar to the yeast his3 promoter, MHC class I TSS selection is regulated: upstream start sites are preferentially utilized under constitutive conditions whereas activated conditions also utilize downstream TSS [36]. Thus, selective use of TSS may be a mechanism to regulate expression under different conditions.

To delineate the role of the core promoter in these differing regulatory environments, we previously characterized elements within the core promoter of the MHC class I gene. The isolated segment between −50 bp and +1 bp retains promoter activity. Although sequences similar to canonical TATA and Inr promoter elements and an Sp1 binding site occur within this region, no single element is absolutely required for transcription [36]. Thus, the MHC class I core promoter has a complex architecture in which no single element is essential, consistent with its selective use of multiple TSS across an extended sequence.

In this study, we extended the analysis of the promoter to the region downstream of the major TSS at +1 to determine whether any downstream regulatory elements reside in this segment of the MHC class I promoter. We report that there are two novel downstream regulatory elements with sequence similarities to previously characterized DPE's, DPE-L1 and DPE–L2 and a third element, GLE, with sequence homology to binding sites for the transcription factor GAGA. The two DPE-L elements preferentially enhance TAF1-dependent transcription from TSS located at the 5′ end of the cluster of multiple start sites in the class I promoter, while GLE increases transcription from all TSSs. We discuss the possible mechanisms by which these downstream elements regulate transcription.

## Results

### The MHC class I promoter contains novel downstream promoter elements

**Figure 1:**
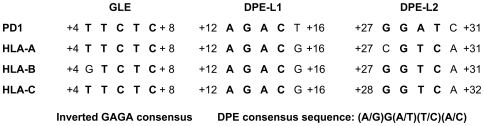
Three downstream sequences are conserved among MHC class I promoters. The sequences +4 to +8 and +12 to +16 and +27/28 to +31/32 of the swine SLA class I gene, PD1, and the human HLA class genes, HLA-A, HLA-B and HLA-C were aligned. All four have conserved GAGA factor binding site and DPE consensus sequences.

Downstream promoter elements (DPE) are conserved among metazoans with a consensus sequence of (A/G)G(A/T)(T/C)(A/C) and are located between +28 to +32 bp downstream of transcription start sites. Examination of four different MHC class I gene sequences identified a consensus GAGA factor binding site at +4 and two consensus DPE sequences at +12 and +27 bp that are conserved in all four promoters (Figure 1), suggesting that these elements may contribute to class I core promoter function.

**Figure 2:**
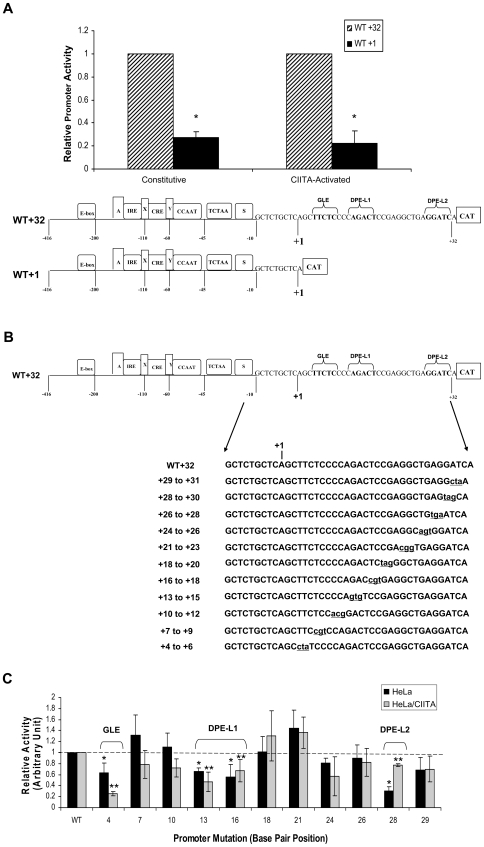
Three downstream elements reside in the downstream region of the MHC class I promoter. **A) Sequences downstream of +1 regulate class I promoter activity in both constitutive and activated transcription.** CAT reporter constructs (5 ug) extending from −416 bp to either +1 (WT+1) to or +32 bp, (WT+32) (see diagram) were transfected into either HeLa cells or HeLa/CIITA cells that stably express CIITA. Promoter activity was assessed by the level of CAT activity as described in Materials and Methods. (*)- denotes a significant (p<0.05) difference between the activities of WT+1 and WT+32, as determined by T-test. This experiment is representative of three independent experiments, each done in duplicate independent transfections. Error bars indicate standard deviation. The absolute levels of MHC class I promoter activity cannot be compared between the HeLa and HeLa/CIITA cell lines due to endogenous CIITA activation of class I promoter activity in the HeLa/CIITA cells. The effect of CIITA in absolute levels of MHC class I promoter activity is shown in the Supplemental Figure S2. **B) Schematic illustration of scanning mutations.** Downstream promoter region mutations were generated in sequential 3 bp clusters, located between +4 bp and +29 bp, within the context of an extended class I promoter with a 5′ terminus at −416 bp and a 3′ terminus at +32 bp. This promoter segment contains an upstream regulatory region that includes series of enhancer elements, a minimal core promoter and the downstream promoter region. Promoter mutation constructs were ligated to a CAT reporter to assess relative promoter activity. Mutations are shown in lower case. **C) Promoter mutations identify three functional elements in the downstream region of the class I promoter.** Each of the scanning promoter mutations was transfected into HeLa cells (black) or HeLa/CIITA cells (grey) and promoter activity determined relative to a wild type control as measured by recovered CAT activity as described in Materials and Methods. The graph summarizes the results of 4 separate experiments, each with duplicate independent transfections. Error bars indicate standard deviation. (*) and (**) denote significant (p<0.05) differences between the activities of mutant constructs relative to the wild type in HeLa cells and HeLa/CIITA cells, respectively.

To determine whether any downstream sequences regulate either constitutive or activated MHC class I promoter activity, we compared the activities of promoter constructs that share a common 5′ extended promoter terminus but differ at their 3′ termini by the presence or absence of 32 bp downstream of +1, which contains the DPE-like and GAGA-like elements (Figure 2A, bottom). The activities of the two promoter constructs, ligated to a CAT reporter, were assayed in transient transfections of native HeLa epithelial cells (Figure 2A, left). Relative constitutive promoter activity in HeLa cells is significantly higher in the presence of the 32 bp downstream segment than in its absence, identifying a positively-acting cis element in this interval. The γ-interferon-induced co-activator, CIITA, activates MHC class I promoter activity. In HeLa cells which stably express CIITA (HeLa/CIITA), relative promoter activity also is significantly greater in the construct that extends to +32 bp than in the one that terminates at +1, demonstrating stimulatory activity of the +1 to +32 bp segment in both CIITA-activated and constitutive, transcription (Figure 2A; Figure S2).

To map these putative downstream promoter activities and to determine whether they correspond to either of the DPE-like (DPE-L) sequences or the GAGA-like (GLE) sequence within the +1–32 bp segment, we generated a series of scanning mutations between +1 to +32 bp downstream of the core promoter region (Fig. 2B). All of the mutations were made within the context of an extended promoter from −416 bp to +32 bp that encompasses the endogenous transcription start sites (TSS) and upstream regulatory elements necessary for both constitutive and activated transcription, i.e. Enhancer A, IRE, and RFX/cyclic AMP response element (Fig. 2A, bottom) [27], [30], [33], [41]–[43]. The activity of each of the promoter constructs, ligated to a CAT reporter, was assessed relative to that of the wild type promoter construct (WT+32) in transient transfections of either HeLa or HeLa/CIITA cells (Figure 2C).

In native HeLa cells, mutations across the segments +4 to +6, +13 to +18, and +28 to +30 resulted in significantly reduced promoter activity, indicating the presence of a downstream element in each of these intervals (Figure 2C). The region +4 to +6 (GLE) coincides with a consensus GAGA factor binding sequence, and the regions +13 to +18 (DPE-L1) and +28 to +30 (DPE-L2) are homologous with canonical DPE consensus sequences. Although the region +18 to +22 bp contains a sequence homologous to the previously described MTE enhancer element [20], mutations across this segment do not affect promoter activity reproducibly or significantly.

In HeLa/CIITA cells, the GLE mutation resulted in significantly reduced promoter activity, relative to the wild type (Figure 2C). Interestingly, the extent of this reduction was about 2–3 fold greater than in native HeLa cells. Mutations in DPE-L1 displayed approximately the same extent of reduction of promoter activity in Hela/CIITA cells as in HeLa cells. However, mutations in DPE-L2, which markedly reduced constitutive promoter activity in native HeLa cells, had only a minimal effect on promoter activity in HeLa/CIITA cells.

Taken together, these findings identify three novel downstream promoter elements that function to enhance MHC class I promoter activity. Two elements, DPE-L1 and DPE-L2, have sequence homology with other reported DPEs. Like other DPE elements, DPE-L2 is located approximately 30 bp downstream of an *in vivo* transcription start site at +1 [44]. Interestingly, DPE-L1 is approximately 30 bp downstream of the TATA-like element, another site of transcription initiation *in vivo* (Weissman et al., unpublished observations). Unlike other downstream elements, the function of these downstream elements is context dependent: the GLE element is a stronger enhancer of activated than constitutive transcription. In contrast, whereas DPE-L2 markedly enhances constitutive transcription, it has a smaller effect on activated transcription.

### DPE-L1 and DPE-L2 functions are not additive

**Figure 3:**
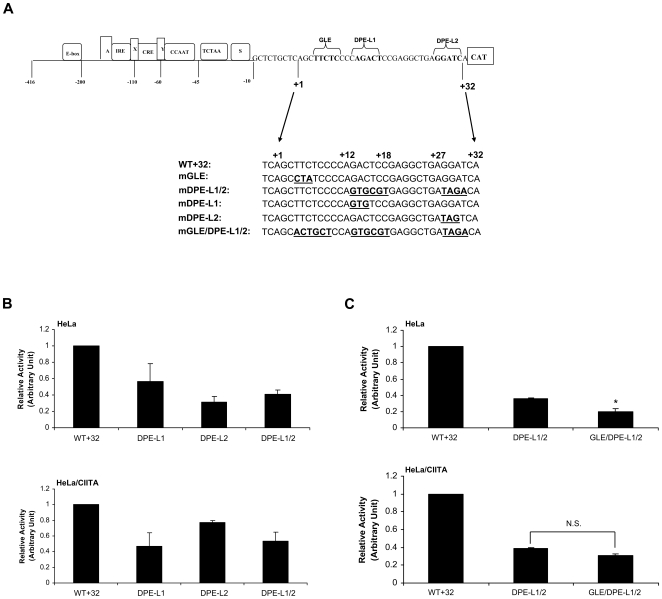
DPE-L1 and DPE-L2 functions are not additive. **A) Schematic illustration of DPE-L1, DPE-L2, GLE, DPE-L1/2 and GLE/DPE-L1/2 mutants.** Double and triple mutations of DPE-L1, DPE-L2, and GLE were designed to encompass the entire region of the each element identified by the scanning mutations. Thus, the mutations in the DPE-L1/2 double promoter mutant extended across +13 to 18 bp and +27 to+30 bp and the GLE/DPE-L1/2 triple promoter mutant included mutations at +4 to +9 bp, +13 to 18 bp and +27 to+30 bp. The single GLE and DPE-L1 and DPE-L2 mutations were the same as shown in Figure 2. **B) The promoter activity of DPE1/2 is indistinguishable from that of either of single DPE mutants.** The two single mutant constructs and the double DPE mutant construct were transfected into HeLa cells or HeLa/CIITA cells and the promoter activity was determined relative to wild type promoter (WT+32). **C) GLE functions independently of DPE-Ls in basal transcription, but not in activated transcription.** The double mutant DPE-L1/2 and the triple mutant GLE/DPE-L1/2 constructs were transfected into HeLa cells or HeLa/CIITA cells and their activities were compared.

Since mutation of any one of the three elements resulted in decreased promoter activity, we next determined whether their activities were additive. To this end, a double mutant spanning both DPE-L1 and DPE-L2 and a triple mutant spanning GLE, DPE-L1, and DPE-L2 were generated (DPE-L1/2 and GLE/DPE-L1/2, Figure 3A). The activity of the DPE-L1/2 promoter mutation was compared to that of the wild type promoter and to the individual DPE-L1 and DPE-L2 mutations in transient transfection assays of both native HeLa cells and HeLa/CIITA cells. If the effect of combining the two mutations was additive, it would suggest that the two elements function independently of each other. As shown in Figure 3B, the activity of the double mutant was not markedly less than either single DPE mutant, either in HeLa or HeLa/CIITA cells. Therefore, the effects of DPE-L1 and DPE-L2 are not additive, indicating that they do not function independently in enhancing either constitutive or activated transcription. Thus, DPE-L1 and DPE-L2 may be sub-elements of a single DPE, as described for DCEs [17].

The activity of the triple GLE/DPE-L1/2 promoter mutation was compared to that of the DPE-L1/2 promoter in transient transfection assays of both native HeLa cells and HeLa/CIITA cells. As shown in Figure 3C (upper panel), in native HeLa cells, the activity of the GLE/DPE-L1/2 promoter is appreciably lower than that of the DPE-L1/2 promoter, suggesting that GLE functions independently of DPE-L1 and DPE-L2 in supporting constitutive transcription from the MHC class I promoter. Surprisingly, in HeLa/CIITA cells, mutation of the GLE element in the context of the DPE-L1/2 mutation does not affect promoter activity (Figure 3C, lower panel). (The activities of double mutations of GLE and either DPE-L1 or DPE-L2 are indistinguishable from that of the triple GLE/DPE-L1/2 mutation in either HeLa or HeLa/CIITA cells (Figure S3)). Thus, the activities of GLE and DPE-L1/2 are context-dependent: GLE functions independently of DPE-Ls in constitutive, but not activated, transcription.

### Downstream elements are necessary for optimal transcription *in vitro*

**Figure 4:**
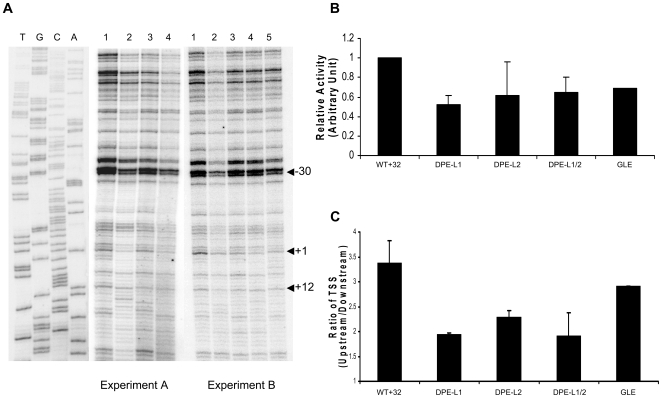
Downstream element mutations affect MHC class I promoter activity *in Vitro*. **A) Mutation of the downstream elements decreases MHC class I promoter activity** ***in vitro*** **.** *In vitro* transcription assays were performed with HeLa nuclear extract and a DNA template consisting of the MHC class I promoter fused to the CAT reporter gene which extended from −416 bp of the class I promoter to the junction with the CAT gene at +32, and continued another 68 bp into the CAT gene. RNA product was assessed by primer extension. DNA sequence ladder is shown on the left. (1) WT+32 wild type promoter; (2) DPE-L1; (3) DPE-L2; (4) DPE-L1/2; (5) GLE (See schematic in Figure 3A). Specific transcription initiation occurs at multiple sites both *in vivo* and *in vitro* [44]. Experiment A and B refer to two independent, replicate experiments. **B) Mutation of the downstream elements reduces promoter activity.** The promoter strength of the wild type, GLE, DPE-L1, DPE-L2, and DPE-L1/2 constructs was analyzed by densitometry of total TSS (−42 bp to +31 bp) to compare total activity of promoter in each construct. **C) Downstream elements preferentially regulate transcription start sites in the upstream region of the promoter.** Analyses of relative transcription start site usage by the wild type, GLE, DPE-L1, DPE-L2, and DPE-L1/2 were done by densitometric quantitation, calculating the ratio between upstream TSS (−42 bp to +2 bp)and downstream TSS (+3 bp to +32 bp). This experiment is representative of two independent experiments.

As previously described, MHC class I transcription initiates at multiple sites within an extended core promoter [36], with major start sites at +1 and +12 and around −30 bp both in vitro and in vivo (Figure 4A). To determine whether the GLE and DPE-L activities observed in the transient transfections reflect direct effects on transcription and, if so, which start sites are affected, the relative promoter activities of GLE, DPE-L1, DPE-L2 and DPE-L1/2 were examined using *in vitro* transcription assays with nuclear extracts from HeLa cells. All three promoter mutants were quantitatively less active *in vitro* than the wild type control promoter in HeLa extracts (Figure 4B), demonstrating that the three elements directly affect transcription. In HeLa/CIITA nuclear extracts, the DPE-L2 mutant promoter construct, unlike the DPE-L1 and GLE mutants, was not less active than the wild type promoter (data not shown), again consistent with the reduced effect of DPE-L2 on CIITA-dependent promoter activity *in vivo*.

Interestingly, quantitative analysis of relative start usage by the wild type, DPE-L1, DPE-L2 and DPE-L1/2 promoters in the *in vitro* transcription assay with HeLa nuclear extract revealed a differential effect on upstream, relative to downstream, start sites. Calculating the ratio between upstream TSS and downstream TSS shows that the DPE-Ls have a preferential effect on upstream TSS, while GLE reduces the overall transcription activity without having significant preferential effect on upstream TSS (Figure 4C).

Thus, the downstream elements, DPE-L1 and DPE-L2, but not GLE, appear to differentially regulate *in vitro* transcription start sites in the upstream versus downstream regions of the promoter. This result is consistent with the observation that GLE and DPE-Ls function independently in the constitutive transcription.

### DPE-L elements preferentially affect constitutive upstream transcription start site selection *in vivo*

**Figure 5:**
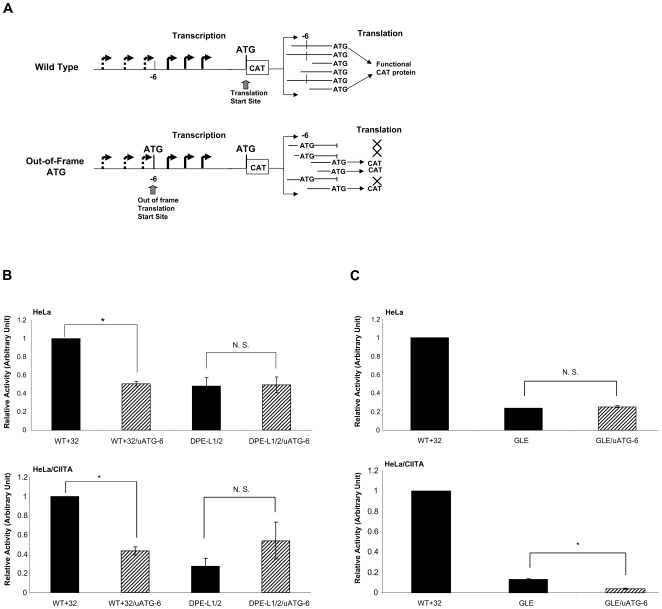
Downstream elements preferentially regulate upstream transcription start sites in constitutive transcription. **A) Schematic of the effect that an out-of-frame ATG insertion has on subsequent translation of mRNA.** Transcription in the class I promoter starts at multiple sites over a span of approximately 60–70 bp [44]. In the wild type promoter, ligated to the CAT reporter, the first ATG encountered by any of these transcripts is the authentic in-frame translation initiation codon that generates functional CAT protein (upper panel). Insertion of an ATG at the −6 bp position results in out-of-frame translation of any mRNAs that initiated upstream of −6 and abortive protein synthesis, whereas translation of transcripts that initiated downstream of −6 bp initiates from the authentic ATG and generates CAT enzyme. Thus, the out-of-frame ATG allows transcripts initiating downstream of −6 bp to be distinguished from those starting upstream. (The indicated start sites are conceptual, to illustrate the strategy, and not intended to denote actual start sites.) The complete strategy and characterization are described in the Results section and [41]. **B) DPE-Ls preferentially target transcription initiating at upstream sites in both constitutive and activated transcription.** HeLa cells (upper panel) or HeLa/CIITA cells (lower panel) were transfected with either a wild type (WT+32) construct; one with an out-of-frame ATG created at −6 bp (WT+32/uATG^−6^); a wild type promoter with mutated DPE-L1 and DPE-L2 (DPE-L1/2); or the out-of-frame ATG promoter construct with mutated DPE-L1 and DPE-L2 (DPE-L1/2/uATG^−6^). The amount of CAT activity relative to the WT+32 was determined as described in Materials and Methods. Whereas the activity of WT+32/uATG^−6^ is significantly differently from WT+32, the activity of DPE-L1/2/uATG^−6^ is not significantly different (N.S.) from that of DPE-L1/2 in either cell line. (*) denotes a significant difference between the activities of WT+32 and WT+32/uATG^−6^. **C) GLE targets upstream start sites in constitutive transcription and downstream start sites in activated transcription.** HeLa cells (upper panel) or HeLa/CIITA cells (lower panel) were transfected with the wild type WT+32, the mutated GLE promoter or the out-of-frame ATG promoter construct with a mutated GLE enhancer, GLE/uATG^−6^. The amount of CAT activity relative to the WT+32 was determined. While GLE/uATG^−6^ is not significantly different from GLE in constitutive transcription in HeLa cells (upper panel), GLE/uATG^−6^ is significantly different from GLE in CIITA-activated transcription in HeLa/CIITA cells (bottom panel). (*) denotes a significant difference between the activities of GLE and GLE/uATG^−6^.

To further assess the differential DPE effects on TSS usage, we next asked whether the DPE's influenced the relative usage of upstream versus downstream start sites *in vivo*. To this end, we employed an *in vivo* translation knock-out strategy which we have characterized extensively previously that distinguishes upstream TSS from downstream ones [36], [44] (schematized in Fig. 5A). The strategy is summarized briefly as follows: A translational out-of-frame ATG (uATG) was generated at the −6 bp position (CTG −> ATG) of the extended core promoter, preserving the overall structural and spatial integrity of the core promoter. The uATG^−6^ is out-of-frame with respect to the translation of the downstream CAT reporter gene. Therefore, translation of transcripts with TSS upstream of −6 bp will initiate at the ATG^−6^, resulting in out-of-frame and abortive translation of the CAT protein product. In contrast, translation of transcripts initiating downstream of −6 bp will initiate at the authentic ATG, be translated normally and generate active CAT protein. Using this strategy, we have demonstrated previously that constitutive transcription initiates primarily at upstream TSS, between −6 and −42 bp. In contrast, CIITA-activated transcription initiates at TSS downstream of −6 bp, at +1 and +12 bp in the wild type promoter [41].

Out-of-frame ATG (uATG) mutations were inserted at −6 bp into the WT, the DPE-L1/2 double mutant and the GLE mutant, transiently transfected into HeLa cells and assayed for CAT activity. Consistent with previous observations that constitutive transcription largely initiates upstream of −6 bp [36], [44], insertion of the uATG into the WT promoter (WT+32/uATG^−6^) resulted in a significant decrease in CAT activity as measured in either transfected HeLa (Figure 5B, upper panel) or HeLa/CIITA cells (Figure 5B, lower panel). (As previously determined, this decrease in activity is not due to the effects of the mutation on transcription nor is the integrity of the wild type −6 nucleotide crucial for expression [36], [44]. In marked contrast, in the context of the double DPE-L1/2 mutations, insertion of the uATG (DPE-L1/2/uATG^−6^) did not significantly reduce production of CAT in either HeLa or HeLa/CIITA cells, beyond the effect of the enhancer mutation itself (Figure 5B). This result indicates that the DPE-L elements do not significantly affect downstream transcription start site usage. Consistent with the decreased upstream TSS usage shown in *in vitro* transcriptions of the DPE-L mutants (Figure 4), these results demonstrate that these downstream elements preferentially regulate start site usage upstream of −6 bp in constitutive transcription.

In contrast, the effect of introducing the GLE mutation in the context of the uATG^−6^ differed when measured in HeLa or in HeLa/CIITA cells. In HeLa cells, CAT activity generated by the GLE/uATG^−6^ construct was not significantly different from that of the GLE construct, consistent with the GLE primarily affecting upstream start sites (Figure 5C, upper panel). However, when the GLE/uATG^−6^ construct was transfected into HeLa/CIITA cells, it generated significantly less CAT activity than the WT+32/uATG^−6^, indicating that during activated transcription the GLE regulates start site usage downstream of −6 bp (Figure 5C, lower panel).

**Figure 6:**
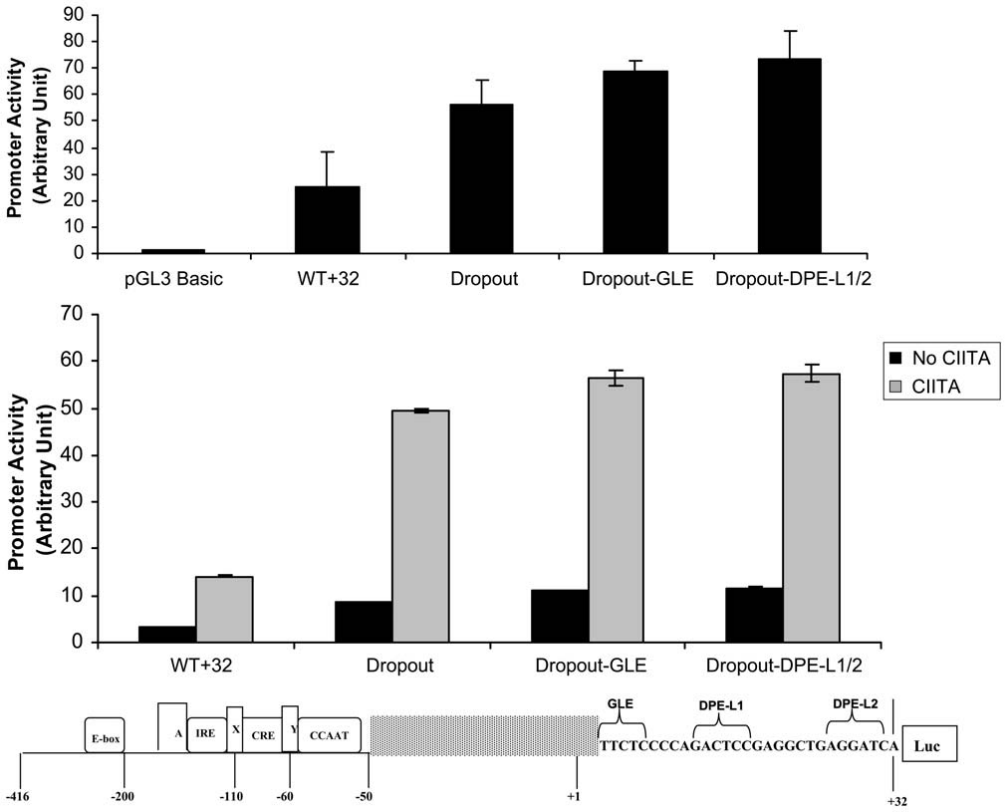
Downstream elements preferentially regulate transcription initiating in the upstream promoter region. HeLa cells were transfected with the wild type dropout construct (5 ug), which has a deletion in the region between −50 and +3 in the context of −416/+32 construct ligated to the luciferase (luc) reporter (see schematic at bottom on Figure), and derivative downstream elements mutant constructs with mutations at GLE and DPE-L1/2 (top). HeLa cells were also co-transfected with a CIITA-expression vector (or a control vector) and the dropout construct or its derivative downstream element mutants (bottom). The amount of luciferase activities was determined as described in Materials and Methods.

In order to further assess preferential regulation of upstream TSS by the downstream promoter elements, we generated a construct from which the upstream transcription start sites between −50 and +3 region were deleted (drop-out); two derivative constructs with mutations in the GLE and DPE-L1/2 were also generated (schematized in Figure 6 bottom). Since these deletion constructs are depleted of the transcription start sites between −50 and +1 bp, the roles of the downstream enhancers on downstream start sites relative to upstream start sites can be assessed directly. The drop-out construct and its derivative mutants were transfected either alone or with a CIITA expression vector into HeLa cells. Surprisingly, the wild type drop-out construct was active and responded to activation by CIITA, despite the removal of the upstream start sites (Figure 6). Indeed, it was consistently more active than the native promoter in both constitutive and activated transcription (Figure 6). This could reflect either that the −50 bp to +3 bp segment negatively regulates downstream promoter activity or that removal of this segment affects promoter activity by altering the distance between an upstream enhancer and the downstream promoter. Thus, the class I promoter contains two core promoter segments each capable of functioning independently; one is located between −50 and +3 bp [44] and the other between +3 and +32 bp. (Deletion of the entire region between −50 and +32 bp results in a construct that is minimally active (Figure S4).)

Importantly, neither GLE nor DPE-L1/2 mutations affect promoter activity in the absence of the transcription start sites located between −50 bp and +3 bp, in either the presence or absence of CIITA. These results extend the conclusion that the downstream DPE-L elements preferentially target transcription start sites clustered at 5′ end of the MHC class I core promoter (Figure 6). Because the GLE mutation does not affect activated transcription, these results also suggest that the GLE element, which affects downstream start sites (Figure 5C) does so indirectly by targeting sequences in the −50 bp to +3 bp interval (see Discussion).

### DPE-L elements preferentially regulate TAF1-dependent, relative to TAF1-independent, MHC class I promoter activity

**Figure 7:**
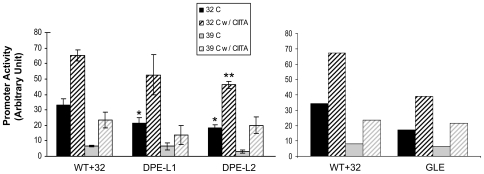
GLE, DPE-L1, and DPE-L2 function differently in TAF1-dependent transcription and TAF1-independent CIITA-mediated transcription. The transcriptional activity regulated by DPE-L1 and DPE-L2 (Left panel) and GLE (Right panel) depend on a functional TAF1. tsBN462 cells were co-transfected at the permissive temperature (32°C)with a CIITA-expression or control vector (which are not TAF1-dependent) and either wild type (WT+32), DPE-L1, DPE-L2 or GLE constructs. All constructs shared common 5′ and 3′ termini at −416 and +32, respectively. After 24 hours, cells were either shifted to the non-permissive temperature (39°C) or left at 32°C for an additional 24 hours prior to harvesting. Promoter activity was assessed by CAT activity as described in Materials and Methods. The results are the representative of two separate sets of independent experiments, each done with independent duplicate transfections. (*) and (**) denote significant differences between the activities of mutant constructs relative to the wild type at 32°C or 39°C with CIITA, respectively.

Unlike constitutive transcription, CIITA-activated transcription of the MHC class I promoter does not depend on the TFIID component, TAF1, as we have shown previously [36], [44]. The requirement for the TAF1 acetyltransferase (AT) activity is by-passed by the intrinsic AT activity of CIITA [45]. Therefore, we next determined whether enhancement of activated class I promoter activity by any of the downstream elements was similarly independent of TAF1. To this end, wild type, GLE, DPE-L1 and DPE-L2 mutant constructs were transfected into tsBN462 cells (Figure 7). These cells have a temperature sensitive mutation in TAF1 which functions normally at the permissive temperature, 32°C, but is inactivated at the non-permissive temperature, 39°C [45]. At the permissive temperature, all of the constructs were active; GLE, DPE-L1 and DPE-L2 mutant constructs were less active than the WT+32. (Since the tsBN462 cells derive from Chinese hamster ovary cells, these results further indicate that the activities of the downstream elements are neither tissue- nor species-specific.) As expected, at the non-permissive temperature, promoter activity of all of the constructs was dramatically reduced to negligible levels, consistent with the dependence of constitutive MHC class I transcription on TAF1.

We could then ask whether the downstream elements regulate CIITA-mediated activation of the class I promoter in the absence of a functional TAF1. To this end, the WT, GLE, and DPE-L mutant promoter constructs were co-transfected into tsBN462 cells with either a CIITA expression vector or control; promoter activity was determined after incubation at either permissive or non-permissive temperatures (Figure 7). At the permissive temperature, the activities of the WT and downstream promoter element mutants were enhanced by CIITA. At the non-permissive temperature, where TAF1 is not functional, CIITA still activated the class I promoter. Importantly, CIITA activated promoter activity is not significantly affected by mutations in the GLE or DPE-L's, relative to the WT promoter. These results indicate that the downstream elements regulate TAF1-dependent, not TAF1-independent, transcription.

### PC4 or CK2 do not mediate DPE-L regulation of MHC class I transcription

The finding that two novel downstream elements, DPE-L1 and DPE-L2, regulate class I promoter activity led to the question of what transcription factors interact with these elements. Studies by Lewis et al. demonstrated that the transcription factors PC4 and CK2 are required for DPE activity [23]. To examine whether either factor plays a role in the DPE-mediated activation of the class I promoter, we asked whether depletion of PC4 or CK2 from HeLa nuclear extracts would alter transcription from an MHC class I promoter template. HeLa nuclear extracts were immunodepleted of either PC4 or CK2 and the depleted extracts analyzed by Western Blot to assess the efficiency of depletion (Supplemental Figure S1A). Immunodepletion removed each factor without affecting *in vitro* transcription from the adenovirus major late promoter (Ad-MLP), which is PC4 independent (data not shown). Immunodepleted extracts were first tested for their ability to support *in vitro* transcription of the wild-type class I promoter template. Depletion of either PC4 or CK2 resulted in a markedly reduced level of in vitro transcription (Supplemental Figure S1B). Transcriptional activity of PC4 depleted extracts could be reconstituted by the addition of exogenous recombinant PC4 (Supplemental Figure S1C). Thus, both PC4 and CK2 regulate class I promoter activity. If either factor targeted one of the DPE-L elements, then depletion of PC4 or CK2 from the HeLa extract would not affect the *in vitro* transcription of the DPE-L mutants. However, the activity of the DPE-L1 and DPE-L2 mutant promoters was as reduced as the WT upon depletion of PC4 or CK2 from extracts (Supplemental Figure S1B). Thus, although depletion of PC4 and CK2 affects class I promoter activity, neither targets either DPE-L.

In previous studies, characterization of the transcription factors that directly interact with the DPEs associated with other promoters revealed that the TAF6/TAF9 heterodimer binds to the DPE [21]. Since the class I DPE-L elements share a high sequence homology, we assessed whether TAF6/TAF9 also binds to the DPE-L elements. In contrast to the binding by canonical DPE, neither DPE-L element – alone or in combination – stably interacts with TAF6/TAF9, either in nuclear extracts or as purified recombinant proteins (data not shown).

The effect of GAGA factor on class I promoter activity was assessed by co-transfecting a GAGA expression vector and either the WT+32 or GLE construct into HeLa cells. Expression of GAGA factor in HeLa cells affects the activity of the wild type promoter (WT+32) but not that of the GLE mutant, consistent with GAGA functioning through the GLE element. Paradoxically, and for reasons that remain to be determined, GAGA factor represses promoter activity through the GLE (Supplementary Figure S5).

## Discussion

MHC class I expression is regulated by at least two distinct categories of pathways: 1) tissue-specific pathways that establish a baseline, or constitutive, level of transcription in any given tissue and 2) dynamically modulated pathways that increase or decrease expression in response to hormonal or cytokine mediated stimuli. The complexity of this regulatory system is reflected in the diversity of regulatory elements associated with the extended class I promoter, the complexity of the core promoter structure and the regulated use of multiple transcription start sites within the core promoter [29], [36], [46], [47]. The complexity of the regulatory mechanisms governing MHC class I transcription is further compounded by the differing activator and general transcription factor requirements of constitutive and activated transcription: Constitutive transcription requires the enzymatic activity of TAF1, whereas activated transcription, as defined by the IFN-γ-induced co-activator CIITA, is TAF1-independent. Thus, class I transcription is regulated by distinct pathways that converge on the core promoter.

In the present study we have characterized three novel MHC class I downstream promoter elements that significantly contribute to the regulation of MHC class I expression. Two of these elements, DPE-L1 and DPE-L2, have sequence similarity to previously described downstream promoter elements, DPE. The third element, the GLE, is homologous to GAGA factor binding sequences. All three elements regulate core promoter activity and preferentially affect transcription start sites clustered at the 5′ end of the core promoter in both constitutive and activated MHC class I transcription. However, their activities appear to be context-dependent, since the relative magnitude of their effects differs in constitutive and activated transcription and they preferentially regulate TAF1-dependent, relative to TAF1-independent, transcription.

DPE-L1 and DPE-L2 show superficial similarities with the canonical DPE. First, both DPE-L1 (AGACT) and DPE-L2 (GGATC) sequences are similar to the canonical DPE sequence ((A/G)G(A/T)(T/C)(A/C)). The positioning of DPE-L2 at +27 bp, relative to the major transcription start site, is similar to that of the DPE at +30 bp. The location of DPE-L1 at +12 to +16 bp places it approximately 30 bp downstream of an upstream start site. Second, the class I promoter, like many DPE-regulated promoters, is TATA-less and TFIID-dependent during constitutive transcription [15]–[17].

Despite their apparent similarities, the class I associated DPE-L1 and DPE-L2 are functionally and mechanistically distinct from previously described DPE elements. Although both DPE and DPE-L function to enhance transcriptional activity, the DPE-L elements differ by targeting a subset of transcription start sites in the upstream promoter region [13], [23]. DPE-L activity is also context dependent, exerting a greater effect in constitutive than activated transcription. The DPE-L elements differ from other described downstream elements in their transcription factor requirements. The transcription factors, CK2 and PC4, which mediate the function of the human DPE, do not mediate DPE-L function, although they do contribute to the overall constitutive transcriptional activity of the class I promoter *in vitro* [23].

Furthermore, the TAF6/TAF9 complex, which mediates recruitment of PIC at the promoter by binding to the canonical DPE [21], does not bind to the DPE-L elements (data not shown). Thus, the transcription factors with which the DPE-L elements interact remain to be identified.

**Figure 8:**
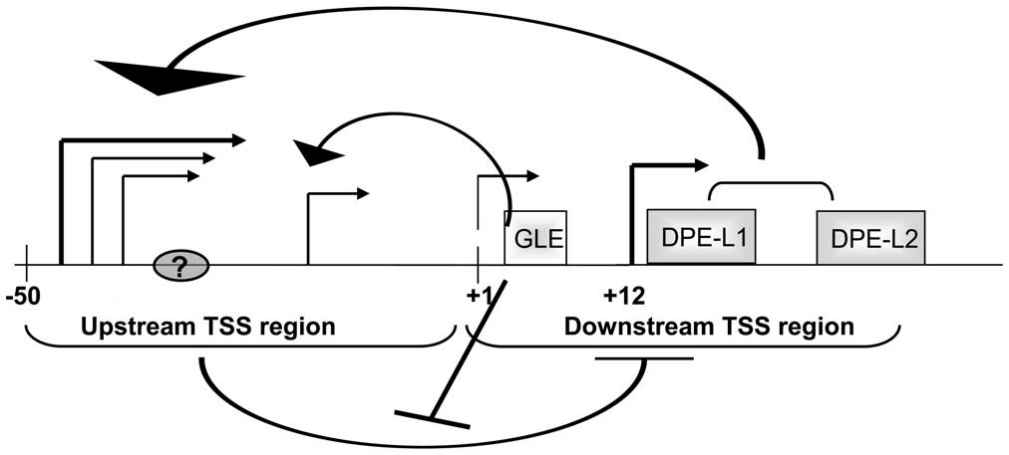
Model of MHC class I gene transcriptional regulation by novel downstream elements. In this model, GLE, DPE-L1, and DPE-L2 regulate the activity of transcription initiating at the upstream region of the promoter (upstream TSS region). GLE and DPE-Ls play different roles: while DPE-Ls enhance transcription from the upstream start sites (arched backward arrows), GLE inhibits the negative regulation of downstream start sites (downstream TSS region) by upstream promoter region (T-shaped symbols), leading to enhancement of transcription activity in a context-dependent manner. Since all three elements target upstream start sites, their activities are context- dependent: in activated transcription, where downstream start site usage is increased, they have less of an effect. Whether the observed negative regulation by the upstream promoter region reflects promoter competition by the upstream start sites (right-angle forward arrows) or the presence of an active negative regulatory element (indicated by shaded ellipse with “?”) remains to be determined.

The function of the GLE is more complex than that of the DPE-L elements. Mutation of GLE leads to decreases in both upstream and downstream transcription start site usage *in vitro*. Furthermore, mutation of GLE in the context of the ATG^−6^ mutant, which only monitors start sites downstream of −6 bp, reduces promoter activity. These results suggest that the GLE affects downstream start site usage. However, paradoxically, the GLE mutation has no effect on activity of the drop-out promoter which contains only downstream start sites. As detailed below and schematized in Figure 8, we propose that the GLE regulates downstream initiation through an upstream target.

The core promoter serves as the molecular platform where regulatory signals delivered by upstream silencer and enhancer elements are integrated [2], [6], [24], [26], [48]. Although core promoter elements were originally defined as only the TATA box and Inr, it is now clear that many core promoters have neither. Many of these promoters, the MHC class I promoter among them, define a novel class of ATG desert promoters that support multiple transcription start sites [23]. The MHC class I promoter consists of two core promoter segments, each of which is capable of supporting transcription independently (Figure 6) [44]. Although the MHC class I core promoter upstream region contains sequences similar to the TATA and Inr elements, neither of them is required for promoter activity [20]. Surprisingly, deletion of the entire promoter segment −50 to +3 bp, which contains the TATA-like and Inr sequences results in enhanced promoter activity. This finding can reflect either competition between the two core promoter regions or the presence of a negative regulatory element in the −50 to +3 bp region. Furthermore, in the absence of the −50 to +3 bp segment, the downstream elements no longer function. Thus, GLE, DPE-L1 and DPE-L2 define a novel set of downstream regulatory elements that regulate upstream promoter activity in the absence of canonical core promoter elements.

Based on these observations, we propose a model in which upstream sequences, located between −50 and +3 bp, negatively regulate downstream promoter activity and the downstream GLE, DPE-l and DPE-L2 elements regulate the activity of the upstream sequences (Figure 8). Specifically, DPE-L1 and DPE-L2 augment transcription from the upstream start sites, whereas the GLE inhibits the negative regulation of downstream promoter activity. This model is consistent with the observations that 1) DPE-L mutations primarily affect upstream start sites, 2) deletion of the upstream core promoter region results in increased transcription from downstream start sites and 3) the GLE mutation affects downstream transcription start site usage in the ATG^−6^ mutant, but not in the −50 to +3 bp drop-out.

Regulatory mechanisms governing transcriptional activation are generally thought to be limited to the recruitment of transcription factors to upstream enhancer and silencer DNA binding sites, which in turn target a set of general transcription factors and co-activators to a core promoter which serves only as a scaffold for transcriptional machinery recruitment. However, it is now evident that the core promoter plays an active role in integrating signaling pathways [41], [49], [50]. The mechanisms that contribute to core promoter element specificity, linked with differential transcription factor usage create a more complex and dynamic layer of regulation mediated by the core promoter itself [26]. The MHC class I core promoter provides a clear example of the active role that core promoters play in integrating regulatory signals. What distinguishes the MHC class I promoter from many other previously studied promoters is the number of converging synergistic and competing signaling pathways that must be integrated to ensure continued immune surveillance in the face of intra-cellular pathogens. In this study, we have identified three novel downstream elements that regulate MHC class I gene expression by integrating regulatory signals on specific transcription start sites. We suspect once other complex mammalian promoters are examined carefully, additional novel regulatory factors and mechanisms will be revealed that will further our understanding of this intricate process.

## Materials and Methods

### Cell Lines and cultivation

The HeLa epithelial, baby hamster kidney (BHK) and tsBN462 cell lines were grown in Dulbecco modified Eagles medium supplemented with 10% fetal bovine serum, 2 mM l-glutamine, 20 mM HEPES (pH 7.2) and gentamicin sulfate (10 ug/mL). Cell lines were maintained in a humidified incubator at 37°C in 7.5% CO_2_, except tsBN462 which were maintained at 32°C in 7.5% CO_2_. HeLa-CIITA cells were provided by Drs. Paul Roche (NIH) and Dr. Peter Cresswell (Yale University).

### Plasmids and cloning strategies

The MHC class I promoter used in these studies was derived from the swine class I gene, PD1 [51], [52]. The PD1 promoter from −416 to +32 bp was ligated to the chloramphenicol acetyltransferase (CAT) reporter DNA that contains a 29 bp 5′ untranslated region, the 1102 bp CAT gene and an 86 bp 3′ untranslated region as previously described (WT+32) [53], [54]. To generate scanning mutants, WT+32 was digested with Blp1 and HindIII (New England Biolabs), followed by ligation to each double stranded oligonucleotide (Integrated DNA Technologies). The BlpI site is located at position −2 within the class I promoter and the HindIII site is located at +32, immediately 5′ to the CAT reporter sequence. The sense strand sequences of the oligonucleotides synthesized (from −2 to +32 bp) are illustrated in Fig. 1A and Fig. 2A. Sequences of scanning mutations were chosen to preserve the GC composition of the sequence but to avoid introducing known regulatory elements. The mammalian expression vector, pcDNA-CIITA was previously described [37]. To generate uATG^−6^ constructs (WT+32/uATG^−6^, DPE-L1/2/uATG^−6^, and GLE/uATG^−6^), a translational out-of-frame ATG (uATG) was inserted at the −6 bp position (CTG −> ATG) of the extended core promoter (WT+32 CAT) as previously described [41]. Dropout constructs were generated by ligating two fragments produced by KpnI/HindIII or KpnI/SfoI digestions of WT+32 Luc, respectively, with an oligonucleotide spanning +3 to +32 bp (with WT, GLE mutant and DPE-L1/2 mutant sequences). GAGA factor-expression vector (pcDNA3-GAGA519) was kindly provided by Dr. Jordi Bernués at the Institut de Biologia Molecular de Barcelona (IBMB-CSIC).

### Transfections

Transient transfections were performed by using a constant amount of DNA (5 ug). At 24 hr prior to transfection 10^6^ HeLa, HeLa/CIITA or tsBN462 cells were seeded in 100-mm tissue culture dishes. Transfections utilized standard calcium phosphate precipitation as previously described [53]. The medium was replaced 24 h after transfection with fresh medium and cells were harvested after an additional 24 h. Temperature-sensitive tsBN462 cells were left at 32°C for 24 h after transfection and then shifted to 39°C (restrictive temperature) or left at 32°C (permissive temperature) for an additional 24 h [45]. HeLa cells were maintained at 37°C for 48 hr after transfection. Reporter activity was corrected by cotransfecting an internal control plasmid RSVLuc (500 ng) or protein levels measured by Bradford Assay. All CAT enzyme assays were measured in the linear range; all data are presented as percent (%) acetylation corrected for transfection efficiency as assessed by luciferase activity or for cell recovery by protein level, in co-transfections with pcDNA-CIITA or pcDNA3-GAGA519. Luciferase determinations were made by using a Monolight 2010 luminometer (Analytical Luminescence Laboratory) and corrected for co-transfected internal control plasmid, TK Renilla. Significance was calculated by T-test and required a threshold of p<0.05.

### *In vitro* transcription and coupled primer extension

*In vitro* transcription reaction mixtures contained 2 ug of class I CAT reporter construct, 6 mM MgCl2, 0.8 mM ribonucleoside triphosphates and 30 U HeLa nuclear extract (Promega) in 20 mM HEPES (pH 7.9), 100 mM KCl, 0.2 mM EDTA, 0.5 mM dithiothreitol, and 20% glycerol in a total of 25 uL was incubated at 23°C for 60 min. Analysis of the *in vitro* -transcribed RNA was done by primer extension as previously described [45].

### Immunodepletions

250 uL of the Santa Cruz anti-CK2b Mab (SC-12739) or anti-PC4 rabbit serum were conjugated to protein A-agarose beads (Boehringer) as described [23]. 100 uL of the conjugated beads was incubated with 200 uL HeLa nuclear extract for 3 hr at 4°C. This was repeated using a second 100 uL of conjugated beads. *In vitro* transcriptions were as described above using equal amounts of either the parent HeLa extract or its CK2/PC4 depleted derivatives.

### Western Blotting

PC4 and CK2β proteins in HeLa nuclear extract (150 µg) were analyzed by sodium dodecyl sulfate (SDS)-12.5% polyacrylamide gel electrophoresis (PAGE) followed by electrophoretic transfer to nitrocellulose membranes. Membranes were blocked in Blotto A (5% milk, 10 mM Tris-HCl [pH 8.0], 150 mM NaCl) for 12 hr at 4°C. Subsequently, an antiserum directed against either PC4 or CK2b (Santa Cruz Biotechnology) was added and incubated in Blotto A-0.05% Tween 20 for 60 min at room temperature. Blots were washed twice in Tris-buffered saline (10 mM Tris-HCl [pH 8.0], 150 mM NaCl)-0.05% Tween 20. 20 uL of a secondary antibody (anti-rabbit immunoglobulin G horseradish peroxidase-conjugated antibody; Santa Cruz Biotechnology) was added to Blotto A-0.05% Tween 20 and incubated for a further 60 min. Blots were then extensively washed in Tris-buffered saline-0.05% Tween 20; specific proteins were detected by chemiluminescence with SuperSignal substrate (Pierce).

## Supporting Information

Figure S1 **PC4 and CK2 contribute to constitutive transcription, but do not mediate the activity of DPE-Ls.** **A) Immunodepletion of HeLa nuclear extract with anti-PC4 and anti-CK2 antibodies effectively deplete PC4 and CK2.** HeLa nuclear extracts (HeLa NE) were depleted with either anti-PC4 (PC4depNE) or anti- CK2β (CK2βNE). Extracts were probed Western blots with either anti-PC4 or anti- CK2β antibodies. **B) DPE-L mutations do not rescue the requirement for PC4 and CK2.** To examine the class I promoter requirement for PC4 and CK2, *in vitro* transcription assays with class I promoter templates (WT+32, DPE-L2; DPE-L1) in either HeLa nuclear extract, extracts depleted of CK2 (DPE-L2-CK2 dep; DPE-L2-CK2dep), or extracts depleted of PC4 (DPE-L2-PC4 dep; DPE-L2-PC4dep). Arrows indicate major *in vivo* transcription start sites. **C) Depletion of PC4 reduces the activity of a wild type promoter template (WT+32).** Addition of exogenous rPC4 to a PC4-depleted HeLa nuclear extract restores promoter activity *in vitro*. *In vitro* transcription reactions were performed with HeLa nuclear extract depleted of PC4 and reconstituted with increasing amounts of exogenous PC4, as indicated. rPC4, recombinant PC4 added to depleted HeLa nuclear extract; WT+32-PC4-dep: *in vitro* transcription of wild type promoter in PC4-depleted HeLa nuclear extract; WT+32, in vitro transcription of wild type promoter in HeLa nuclear extract; HeLa NE, background transcription of extract in the absence of exogenous DNA.
(TIF)

Figure S2 **Effect of CIITA on absolute level of MHC class I promoter activity.** HeLa cells were co-transfected with a CIITA-expression vector, or control vector, and either the −416/+32 CAT (WT+32) or −416/+1 CAT (WT+1) constructs. Promoter activity was assessed as described in Materials and Methods.
(TIF)

Figure S3 **The promoter activity of the GLE/DPE1/2 triple mutant is indistinguishable from that of GLE/DPE-L double mutants.** The two double GLE/DPE-L mutant constructs and the triple GLE/DPE-L1/2 mutant construct were transfected into HeLa cells (upper panel) or HeLa/CIITA cells (lower panel) and the promoter activity was determined relative to wild type promoter (WT+32), as described in Materials and Methods.
(TIF)

Figure S4 **Transcription from the MHC class I promoter predominantly initiates downstream of −50 bp.** HeLa cells were transfected with either the wild type construct (WT+32), the dropout construct, which has a deletion in the region between −50 and +3 in the context of −416/+32 construct ligated to the luciferase (luc) reporter (Dropout), a 3′ truncation construct deleted of the region −50 to +32(WT-50) or a control vector (pGL3basic) (see schematic at bottom on Figure). Promoter activity was assessed as described in Materials and Methods.
(TIF)

Figure S5 **Effect of GAGA factor on MHC class I promoter activity.** HeLa cells were co-transfected with a GAGA factor-expression vector, or control vector, and either the −416/+32 CAT (WT+32) or GLE constructs. Promoter activity was assessed as described in Materials and Methods.
(TIF)

## References

[1] Wong C, Rougier-Chapman EM, Frederick JP, Datto MB, Liberati NT (1999). Smad3-Smad4 and AP-1 complexes synergize in transcriptional activation of the c-Jun promoter by transforming growth factor beta.. Mol Cell Biol.

[2] Berk AJ (1999). Activation of RNA polymerase II transcription.. Curr Opin Cell Biol.

[3] Chen JL, Attardi LD, Verrijzer CP, Yokomori K, Tjian R (1994). Assembly of recombinant TFIID reveals differential coactivator requirements for distinct transcriptional activators.. Cell.

[4] Gill G (2001). Regulation of the initiation of eukaryotic transcription.. Essays Biochem.

[5] Kadonaga JT (2004). Regulation of RNA polymerase II transcription by sequence-specific DNA binding factors.. Cell.

[6] Roeder RG (1996). The role of general initiation factors in transcription by RNA polymerase II.. Trends Biochem Sci.

[7] Struhl K (2001). Gene regulation. A paradigm for precision.. Science.

[8] Burke TW, Kadonaga JT (1997). The downstream core promoter element, DPE, is conserved from Drosophila to humans and is recognized by TAFII60 of Drosophila.. Genes Dev.

[9] Kutach AK, Kadonaga JT (2000). The downstream promoter element DPE appears to be as widely used as the TATA box in Drosophila core promoters.. Mol Cell Biol.

[10] Lagrange T, Kapanidis AN, Tang H, Reinberg D, Ebright RH (1998). New core promoter element in RNA polymerase II-dependent transcription: sequence-specific DNA binding by transcription factor IIB.. Genes Dev.

[11] Lee DH, Gershenzon N, Gupta M, Ioshikhes IP, Reinberg D (2005). Functional characterization of core promoter elements: the downstream core element is recognized by TAF1.. Mol Cell Biol.

[12] Smale ST, Baltimore D (1989). The “initiator” as a transcription control element.. Cell.

[13] Willy PJ, Kobayashi R, Kadonaga JT (2000). A basal transcription factor that activates or represses transcription.. Science.

[14] Butler JE, Kadonaga JT (2002). The RNA polymerase II core promoter: a key component in the regulation of gene expression.. Genes Dev.

[15] Smale ST, Kadonaga JT (2003). The RNA polymerase II core promoter.. Annu Rev Biochem.

[16] Burke TW, Kadonaga JT (1996). Drosophila TFIID binds to a conserved downstream basal promoter element that is present in many TATA-box-deficient promoters.. Genes Dev.

[17] Lewis BA, Kim TK, Orkin SH (2000). A downstream element in the human beta-globin promoter: evidence of extended sequence-specific transcription factor IID contacts.. Proc Natl Acad Sci U S A.

[18] Tokusumi Y, Ma Y, Song X, Jacobson RH, Takada S (2007). The new core promoter element XCPE1 (X Core Promoter Element 1) directs activator-, mediator-, and TATA-binding protein-dependent but TFIID-independent RNA polymerase II transcription from TATA-less promoters.. Mol Cell Biol.

[19] Anish R, Hossain MB, Jacobson RH, Takada S (2009). Characterization of transcription from TATA-less promoters: identification of a new core promoter element XCPE2 and analysis of factor requirements.. PLoS One.

[20] Lim CY, Santoso B, Boulay T, Dong E, Ohler U (2004). The MTE, a new core promoter element for transcription by RNA polymerase II.. Genes Dev.

[21] Shao H, Revach M, Moshonov S, Tzuman Y, Gazit K (2005). Core promoter binding by histone-like TAF complexes.. Mol Cell Biol.

[22] Wright KJ, Marr MT, Tjian R (2006). TAF4 nucleates a core subcomplex of TFIID and mediates activated transcription from a TATA-less promoter.. Proc Natl Acad Sci U S A.

[23] Lewis BA, Sims RJ, Lane WS, Reinberg D (2005). Functional characterization of core promoter elements: DPE-specific transcription requires the protein kinase CK2 and the PC4 coactivator.. Mol Cell.

[24] Hampsey M (1998). Molecular genetics of the RNA polymerase II general transcriptional machinery.. Microbiol Mol Biol Rev.

[25] Reinberg D, Orphanides G, Ebright R, Akoulitchev S, Carcamo J (1998). The RNA polymerase II general transcription factors: past, present, and future.. Cold Spring Harb Symp Quant Biol.

[26] Smale ST (2001). Core promoters: active contributors to combinatorial gene regulation.. Genes Dev.

[27] Girdlestone J (1996). Transcriptional regulation of MHC class I genes.. Eur J Immunogenet.

[28] Le Bouteiller P (1994). HLA class I chromosomal region, genes, and products: facts and questions.. Crit Rev Immunol.

[29] Singer DS, Maguire JE (1990). Regulation of the expression of class I MHC genes.. Crit Rev Immunol.

[30] David-Watine B, Israel A, Kourilsky P (1990). The regulation and expression of MHC class I genes.. Immunol Today.

[31] Girdlestone J (1995). Regulation of HLA class I loci by interferons.. Immunobiology.

[32] Singer DS, Mozes E, Kirshner S, Kohn LD (1997). Role of MHC class I molecules in autoimmune disease.. Crit Rev Immunol.

[33] Gobin SJ, Peijnenburg A, Keijsers V, van den Elsen PJ (1997). Site alpha is crucial for two routes of IFN gamma-induced MHC class I transactivation: the ISRE-mediated route and a novel pathway involving CIITA.. Immunity.

[34] Ishiguro N, Brown GD, Meruelo D (1997). Activation transcription factor 1 involvement in the regulation of murine H-2Dd expression.. J Biol Chem.

[35] Nagarajan UM, Peijnenburg A, Gobin SJ, Boss JM, van den elsen PJ (2000). Novel mutations within the RFX-B gene and partial rescue of MHC and related genes through exogenous class II transactivator in RFX-B-deficient cells.. J Immunol.

[36] Howcroft TK, Singer DS (2003). Expression of nonclassical MHC class Ib genes: comparison of regulatory elements.. Immunol Res.

[37] Martin BK, Chin KC, Olsen JC, Skinner CA, Dey A (1997). Induction of MHC class I expression by the MHC class II transactivator CIITA.. Immunity.

[38] Raval A, Howcroft TK, Weissman JD, Kirshner S, Zhu XS (2001). Transcriptional coactivator, CIITA, is an acetyltransferase that bypasses a promoter requirement for TAF(II)250.. Mol Cell.

[39] Iyer V, Struhl K (1995). Mechanism of differential utilization of the his3 TR and TC TATA elements.. Mol Cell Biol.

[40] Carninci P, Sandelin A, Lenhard B, Katayama S, Shimokawa K (2006). Genome-wide analysis of mammalian promoter architecture and evolution.. Nat Genet.

[41] Lee MP, Howcroft K, Kotekar A, Yang HH, Buetow KH (2005). ATG deserts define a novel core promoter subclass.. Genome Res.

[42] Singer DS, Ehrlich R (1988). Identification of regulatory elements associated with a class I MHC gene.. Curr Top Microbiol Immunol.

[43] Raval A, Weissman JD, Howcroft TK, Singer DS (2003). The GTP-binding domain of class II transactivator regulates its nuclear export.. J Immunol.

[44] Howcroft TK, Raval A, Weissman JD, Gegonne A, Singer DS (2003). Distinct transcriptional pathways regulate basal and activated major histocompatibility complex class I expression.. Mol Cell Biol.

[45] Weissman JD, Howcroft TK, Singer DS (2000). TAF(II)250-independent transcription can be conferred on a TAF(II)250-dependent basal promoter by upstream activators.. J Biol Chem.

[46] Howcroft TK, Murphy C, Weissman JD, Huber SJ, Sawadogo M (1999). Upstream stimulatory factor regulates major histocompatibility complex class I gene expression: the U2DeltaE4 splice variant abrogates E-box activity.. Mol Cell Biol.

[47] Maguire JE, Frels WI, Richardson JC, Weissman JD, Singer DS (1992). In vivo function of regulatory DNA sequence elements of a major histocompatibility complex class I gene.. Mol Cell Biol.

[48] Kaufmann J, Verrijzer CP, Shao J, Smale ST (1996). CIF, an essential cofactor for TFIID-dependent initiator function.. Genes Dev.

[49] Butler JE, Kadonaga JT (2001). Enhancer-promoter specificity mediated by DPE or TATA core promoter motifs.. Genes Dev.

[50] Ohtsuki S, Levine M, Cai HN (1998). Different core promoters possess distinct regulatory activities in the Drosophila embryo.. Genes Dev.

[51] Frels WI, Bluestone JA, Hodes RJ, Capecchi MR, Singer DS (1985). Expression of a microinjected porcine class I major histocompatibility complex gene in transgenic mice.. Science.

[52] Singer DS, Camerini-Otero RD, Satz ML, Osborne B, Sachs D (1982). Characterization of a porcine genomic clone encoding a major histocompatibility antigen: expression in mouse L cells.. Proc Natl Acad Sci U S A.

[53] Howcroft TK, Richardson JC, Singer DS (1993). MHC class I gene expression is negatively regulated by the proto-oncogene, c-jun.. EMBO J.

[54] Howcroft TK, Palmer LA, Brown J, Rellahan B, Kashanchi F (1995). HIV Tat represses transcription through Sp1-like elements in the basal promoter.. Immunity.

