# Supplementary figures and images for: Three Novel Downstream Promoter Elements Regulate MHC Class I Promoter Activity in Mammalian Cells
Source: PLoS One. 2010 Dec 13;5(12):e15278. doi: 10.1371/journal.pone.0015278 (PMC3001478; doi:10.1371/journal.pone.0015278)

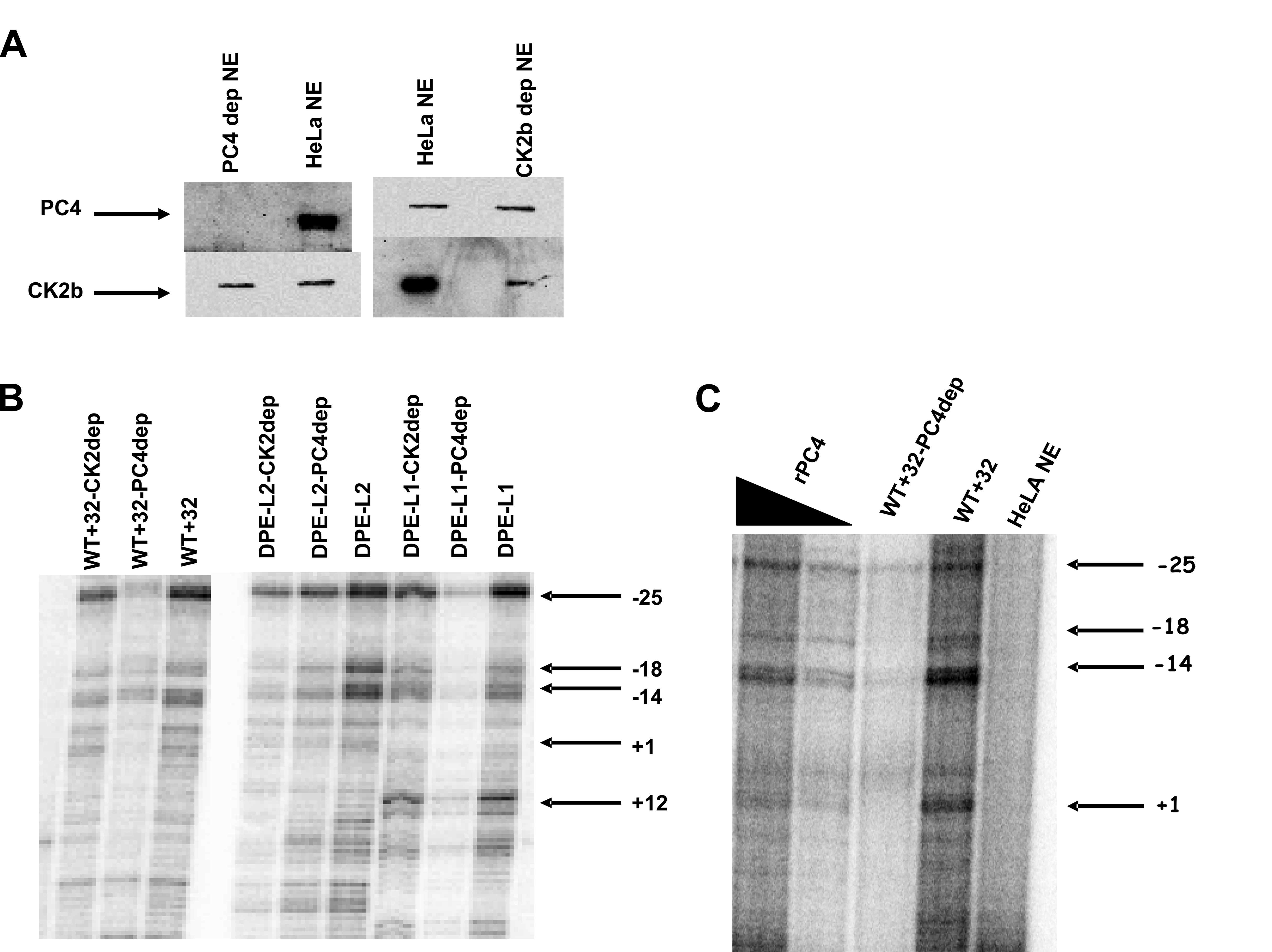

Supplement: Figure S1 — PC4 and CK2 contribute to constitutive transcription, but do not mediate the activity of DPE-Ls. A) Immunodepletion of HeLa nuclear extract with anti-PC4 and anti-CK2 antibodies effectively deplete PC4 and CK2. HeLa nuclear extracts (HeLa NE) were depleted with either anti-PC4 (PC4depNE) or anti- CK2β (CK2βNE). Extracts were probed Western blots with either anti-PC4 or anti- CK2β antibodies. B) DPE-L mutations do not rescue the requirement for PC4 and CK2. To examine the class I promoter requirement for PC4 and CK2, in vitro transcription assays with class I promoter templates (WT+32, DPE-L2; DPE-L1) in either HeLa nuclear extract, extracts depleted of CK2 (DPE-L2-CK2 dep; DPE-L2-CK2dep), or extracts depleted of PC4 (DPE-L2-PC4 dep; DPE-L2-PC4dep). Arrows indicate major in vivo transcription start sites. C) Depletion of PC4 reduces the activity of a wild type promoter template (WT+32). Addition of exogenous rPC4 to a PC4-depleted HeLa nuclear extract restores promoter activity in vitro. In vitro transcription reactions were performed with HeLa nuclear extract depleted of PC4 and reconstituted with increasing amounts of exogenous PC4, as indicated. rPC4, recombinant PC4 added to depleted HeLa nuclear extract; WT+32-PC4-dep: in vitro transcription of wild type promoter in PC4-depleted HeLa nuclear extract; WT+32, in vitro transcription of wild type promoter in HeLa nuclear extract; HeLa NE, background transcription of extract in the absence of exogenous DNA. (TIF) [file pone.0015278.s001.tif]

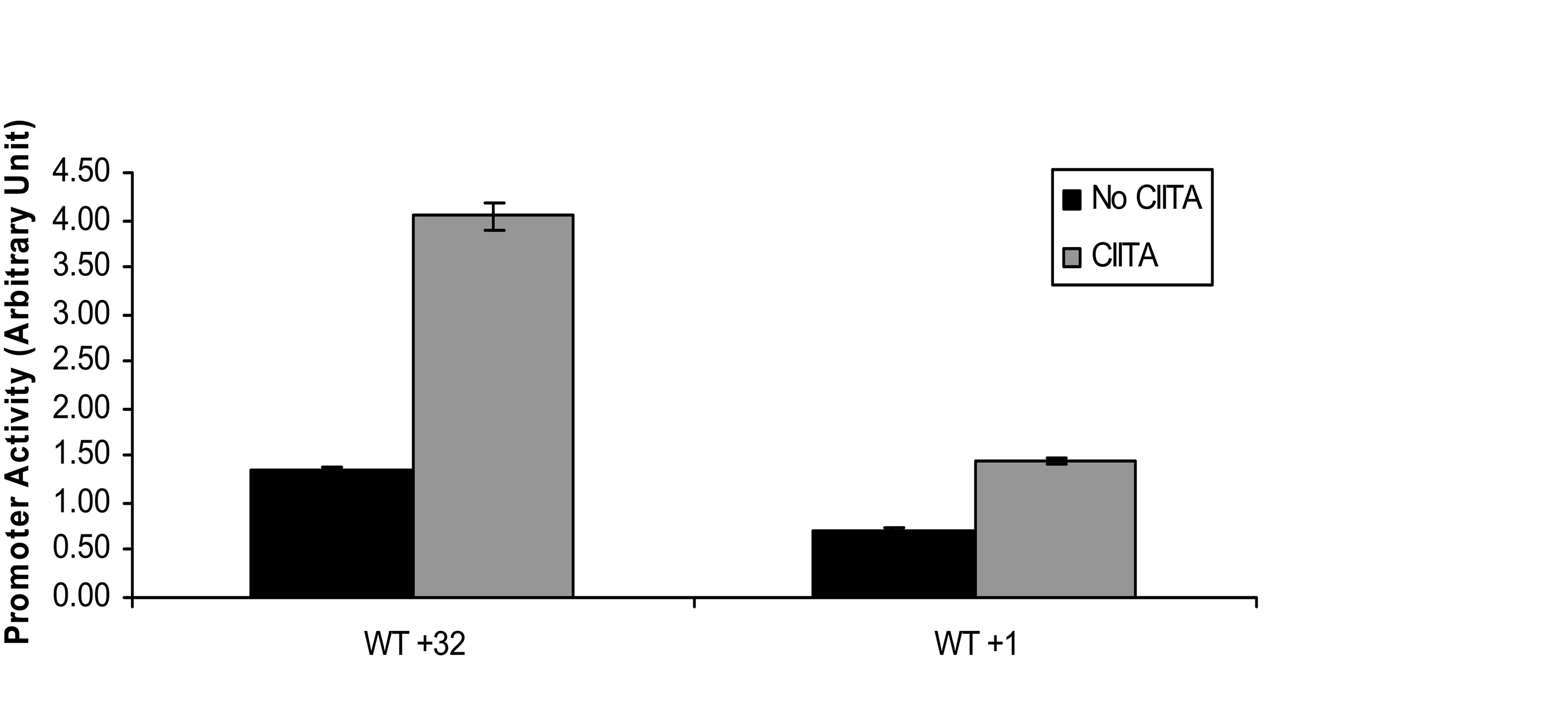

Supplement: Figure S2 — Effect of CIITA on absolute level of MHC class I promoter activity. HeLa cells were co-transfected with a CIITA-expression vector, or control vector, and either the −416/+32 CAT (WT+32) or −416/+1 CAT (WT+1) constructs. Promoter activity was assessed as described in Materials and Methods. (TIF) [file pone.0015278.s002.tif]

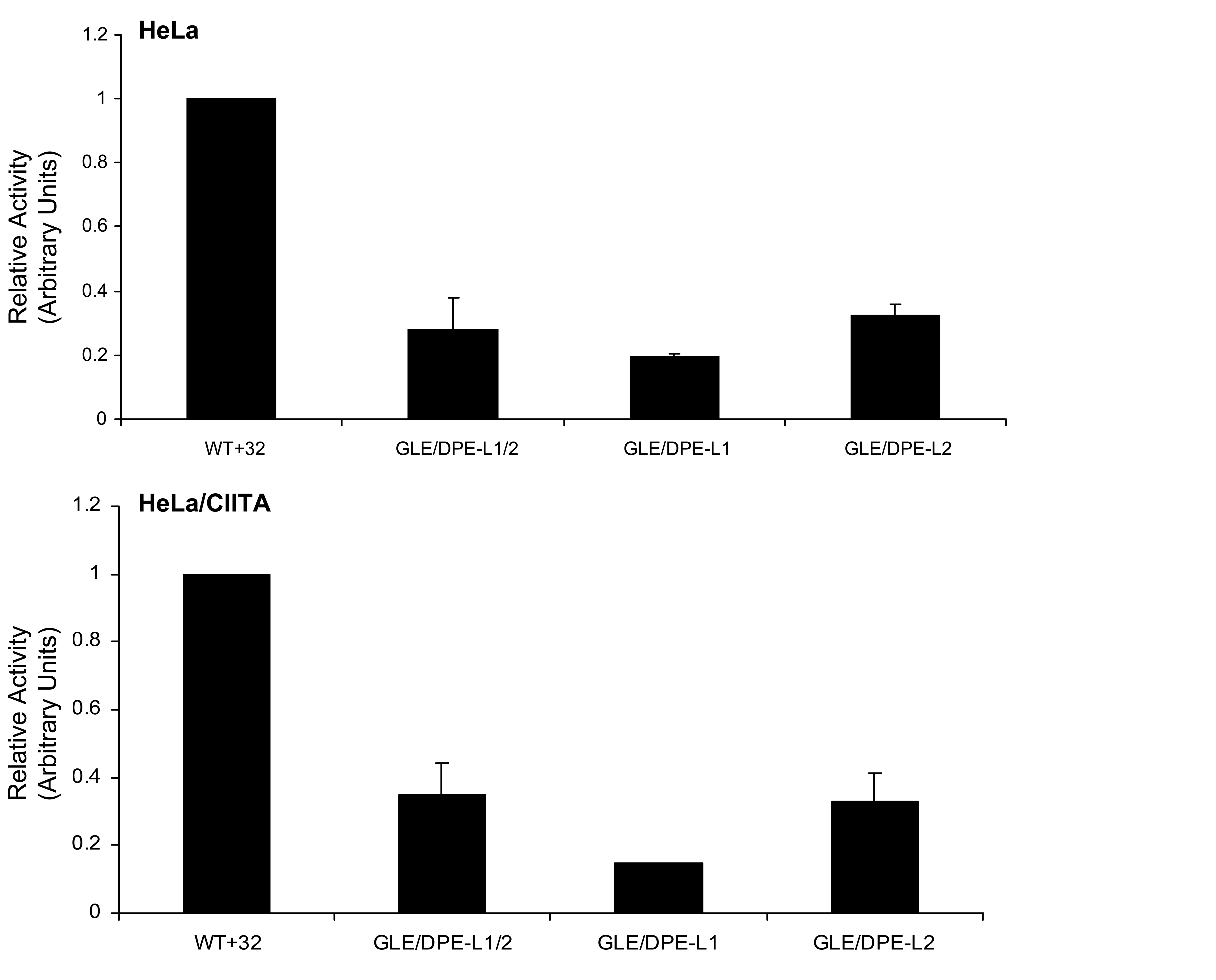

Supplement: Figure S3 — The promoter activity of the GLE/DPE1/2 triple mutant is indistinguishable from that of GLE/DPE-L double mutants. The two double GLE/DPE-L mutant constructs and the triple GLE/DPE-L1/2 mutant construct were transfected into HeLa cells (upper panel) or HeLa/CIITA cells (lower panel) and the promoter activity was determined relative to wild type promoter (WT+32), as described in Materials and Methods. (TIF) [file pone.0015278.s003.tif]

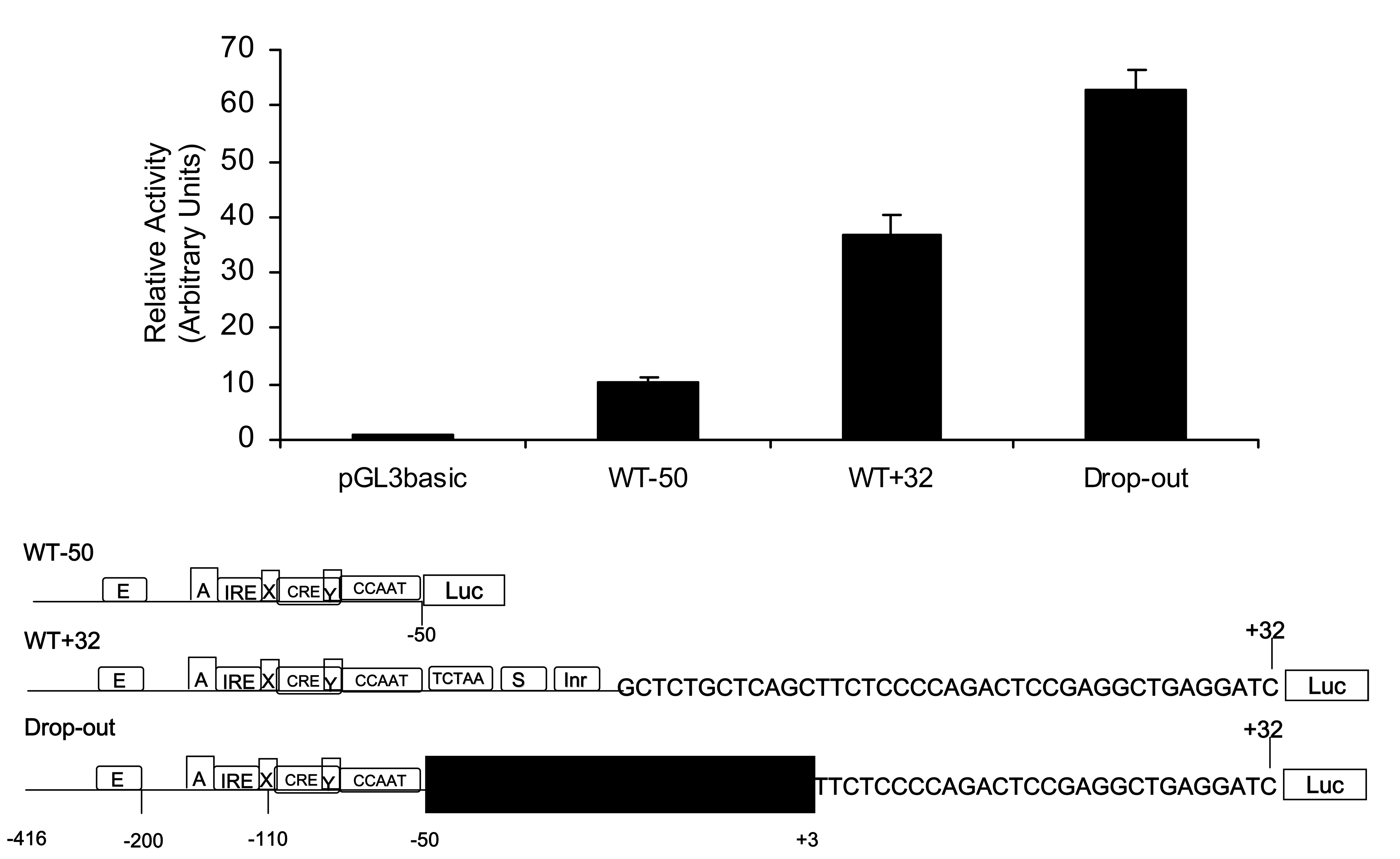

Supplement: Figure S4 — Transcription from the MHC class I promoter predominantly initiates downstream of −50 bp. HeLa cells were transfected with either the wild type construct (WT+32), the dropout construct, which has a deletion in the region between −50 and +3 in the context of −416/+32 construct ligated to the luciferase (luc) reporter (Dropout), a 3′ truncation construct deleted of the region −50 to +32(WT-50) or a control vector (pGL3basic) (see schematic at bottom on Figure). Promoter activity was assessed as described in Materials and Methods. (TIF) [file pone.0015278.s004.tif]

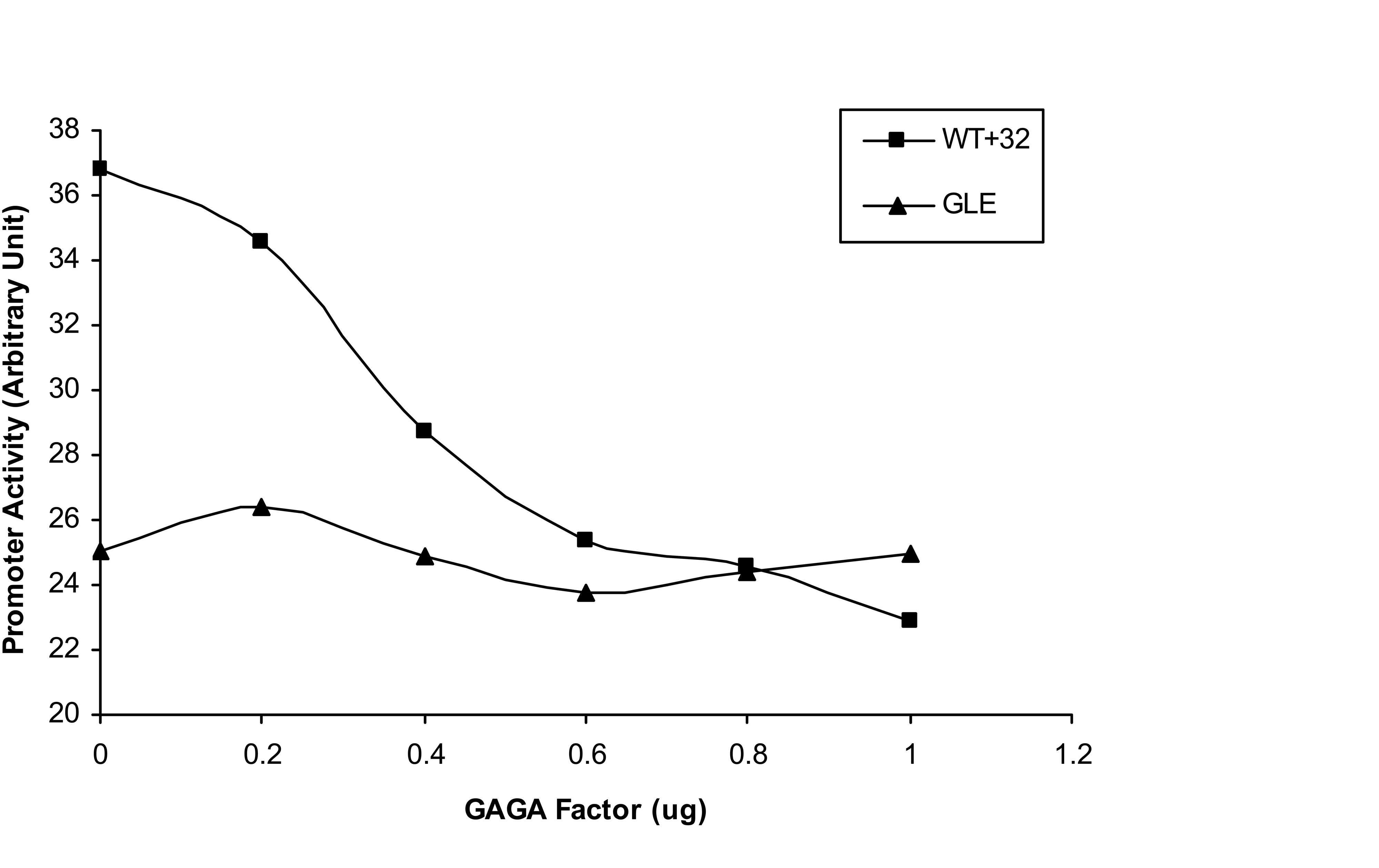

Supplement: Figure S5 — Effect of GAGA factor on MHC class I promoter activity. HeLa cells were co-transfected with a GAGA factor-expression vector, or control vector, and either the −416/+32 CAT (WT+32) or GLE constructs. Promoter activity was assessed as described in Materials and Methods. (TIF) [file pone.0015278.s005.tif]
